# Genotoxicity of Graphene-Based Materials

**DOI:** 10.3390/nano12111795

**Published:** 2022-05-24

**Authors:** Josefa Domenech, Adriana Rodríguez-Garraus, Adela López de Cerain, Amaya Azqueta, Julia Catalán

**Affiliations:** 1 Finnish Institute of Occupational Health, Box 40, Työterveyslaitos, 00032 Helsinki, Finland; josefa.domenech@ttl.fi (J.D.); adriana.rodriguezgarraus@ttl.fi (A.R.-G.); 2 Department of Pharmacology and Toxicology, Faculty of Pharmacy and Nutrition, Universidad de Navarra, Irunlarrea 1, 31008 Pamplona, Spain; acerain@unav.es (A.L.d.C.); amazqueta@unav.es (A.A.); 3 IdiSNA, Navarra Institute for Health Research, Irunlarrea 3, 31008 Pamplona, Spain; 4 Department of Anatomy, Embryology and Genetics, University of Zaragoza, 50013 Zaragoza, Spain

**Keywords:** graphene-based materials, genotoxicity, mutagenicity

## Abstract

Graphene-based materials (GBMs) are a broad family of novel carbon-based nanomaterials with many nanotechnology applications. The increasing market of GBMs raises concerns on their possible impact on human health. Here, we review the existing literature on the genotoxic potential of GBMs over the last ten years. A total of 50 articles including in vitro, in vivo, in silico, and human biomonitoring studies were selected. Graphene oxides were the most analyzed materials, followed by reduced graphene oxides. Most of the evaluations were performed in vitro using the comet assay (detecting DNA damage). The micronucleus assay (detecting chromosome damage) was the most used validated assay, whereas only two publications reported results on mammalian gene mutations. The same material was rarely assessed with more than one assay. Despite inhalation being the main exposure route in occupational settings, only one in vivo study used intratracheal instillation, and another one reported human biomonitoring data. Based on the studies, some GBMs have the potential to induce genetic damage, although the type of damage depends on the material. The broad variability of GBMs, cellular systems and methods used in the studies precludes the identification of physico-chemical properties that could drive the genotoxicity response to GBMs.

## 1. Introduction

**Figure 1:**
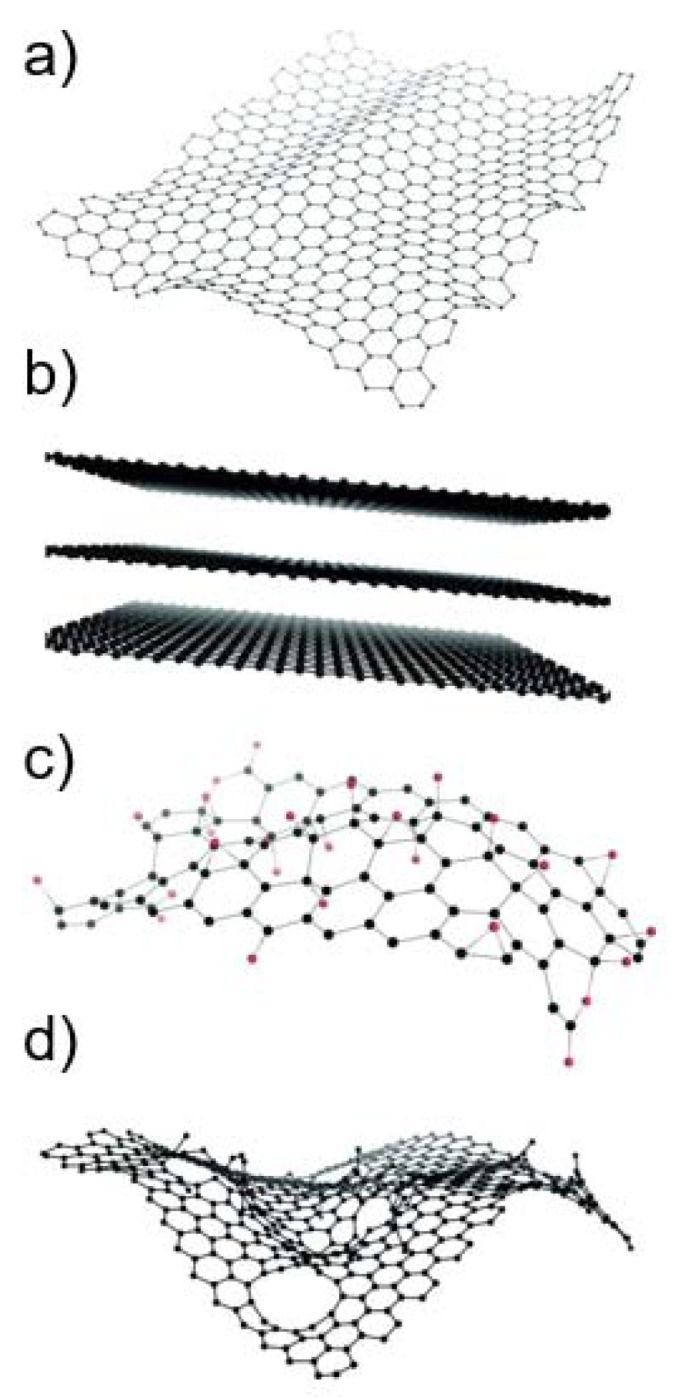
Representative chemical structures of some of the graphene-based materials: (**a**) graphene, (**b**) few-layer graphene, (**c**) graphene oxide (oxygen atoms are in red) and (**d**) reduced graphene oxide. Reprinted with permission from Ref. [1]. Copyright 2013 Wiley Online Library.

Graphene-based materials (GBMs) are a family of novel carbon-based nanomaterials with great potential in many nanotechnology applications [1,2]. Graphene consists of a flat monolayer of carbon atoms arranged in a two-dimensional honeycomb-like structure, with a high surface area on both sides of the planar axis [2,3] (Figure 1a). Due to its monolayer structure, graphene is the only known solid material in which every atom is available for chemical interaction from two sides [4]. It can be wrapped up into buckyballs of fullerenes, rolled into nanotubes, or stacked into graphite [3]. Different GBMs can be obtained by oxidation and/or functionalization of graphene. As GBMs constitute a broad family of materials whose physico-chemical (PC) properties can strongly vary [5], the European Union Graphene Flagship project suggested a classification framework based on three main PC descriptors: the number of graphene layers, the average lateral size, and the carbon-to-oxygen (C/O) ratio [6]. Based on these parameters, different authors [2,7] have proposed classifying GBMs into graphene oxide (GO), reduced graphene oxide (rGO), few-layer graphene (FLG), graphene nanosheets and flakes, and graphene ribbons and dots; some of which are exemplified in Figure 1b–d. In addition, the planar surface of graphene can be functionalized with, e.g., carbonyl, hydroxyl, and epoxy groups, or with capping agents or coatings, such as polyethylene glycol, to make it more compatible with its applications [4].

As already mentioned, the different members of the large family of GBMs are endowed with different PC characteristics, including variable lateral size, thickness, surface area, shape, C/O ratio, and surface chemistry [2,5]. As a result, GBMs exhibit a broad range of extraordinary properties, such as mechanical stiffness, strength, elasticity, as well as high electrical and thermal conductivity, which make them attractive for a multitude of different applications [8]. In fact, GBMs are one of the most promising tools in the development of batteries, supercapacitors, and solar cells [9,10,11]. GBMs are also applied in advanced food packaging, foldable touch screens and superprotective coatings for wind turbines and ships [4], as well as in biomedical applications, such as drug delivery systems, biosensors, anti-bacterial agents, tissue engineering, and imaging systems for example [12,13].

The increasing market and use of GBMs calls for a thorough evaluation of their possible impact on human health [4,8,14]. As reported with other nanomaterials, the different PC properties of graphene and its derivatives guide their interaction with biological systems, and this may affect their toxic response [12]. In fact, previous studies have suggested that the hazard potential of GBMs may vary considerably depending on their different properties, such as size, surface structure, functionalization, charge, impurities, and aggregation/agglomeration state [4,15]. The main risk posed by GBMs to human health appears to be associated with occupational exposure to these materials through inhalation during their production, use, and waste disposal [2]. The few available in vivo inhalation toxicity studies showed pulmonary inflammation, fibrosis, and long GBMs persistence in rodents [2,8,14]. Tentatively, some GBMs might have similar toxic properties to carbon nanotubes, some of which are known to be genotoxic and carcinogenic [14].

One of the main safety concerns related to nanomaterials is their potential genotoxicity [16,17]. Genotoxicity describes any alterations that affect the integrity of genetic material [18]. DNA lesions can still be repaired by cellular repair systems. However, if the lesions are misrepaired or if they remain unrepaired at the time of DNA replication, then they can lead to permanent changes or mutations in the gene sequence (gene mutations) or in the chromosomal structure (chromosomal mutations). In addition, abnormalities in the chromosome number (genomic mutations) can arise as a consequence of the interference of the materials with the mitotic or meiotic apparatus [18]. Mutations occurring in critical genes may lead to cancer [19]. Therefore, every mutagen is considered to be potentially carcinogenic [17]. Furthermore, mutations also play an important role in reproductive and developmental abnormalities [18] and other diseases [20,21].

Due to the important consequences to human health, together with the fact that genotoxic carcinogens are regarded as having no threshold that would allow establishing a non-observed adverse effect level, mutagenicity is a hazard endpoint required in all product regulations, e.g., Registration, Evaluation, Authorization and Restriction of Chemicals (REACH), biocides, medical devices, food additives, cosmetics, etc. [22]. Genotoxicity is also a key point in most of the testing strategies suggested for nanomaterials [16,23,24,25], and its assessment has been highly recommended at early stages of the innovation process [4]. However, as it is for other nanomaterials, some of the standard test guidelines developed by The Organization for Economic Co-operation and Development (OECD) that were validated for soluble chemicals may not be compatible with GBMs [4,16,26]. Furthermore, non-validated assays, such as the in vitro comet assay, can provide relevant information about the genotoxic mechanisms of action of GBMs [17], and on the relationship between the PC properties of these materials and their genotoxic potential. Currently, the comet assay is the most used in vitro technique to evaluate the genotoxicity of nanomaterials at research level [26,27,28]. Therefore, although not yet validated, the European Food Safety Authority (EFSA) genotoxicity guidance to assess the risks of nanomaterials applied in food and feed [23] recommends applying the comet assay to provide information about the genotoxic mechanism of action of these materials.

**Figure 2:**
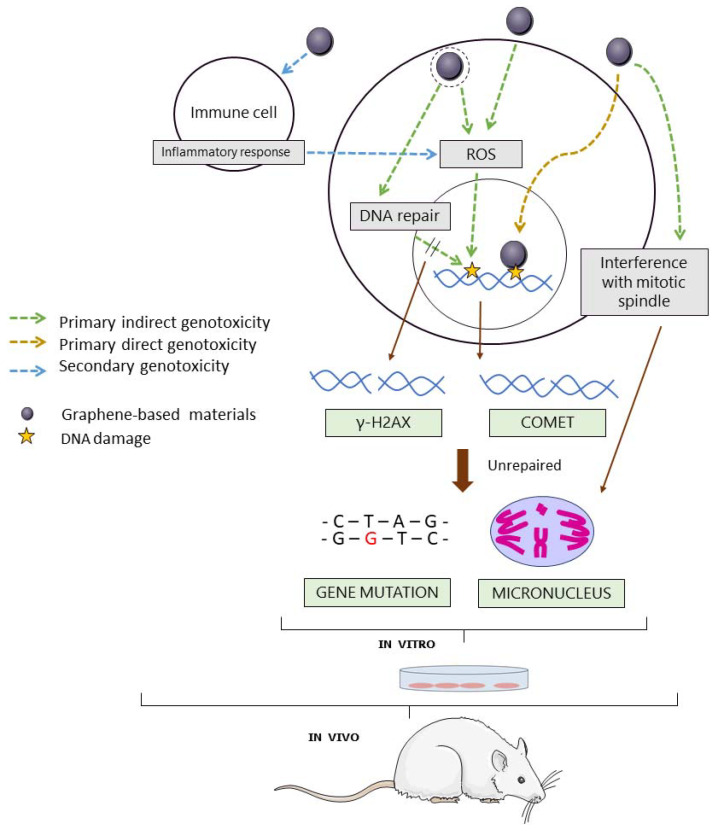
Genotoxic mechanisms of action of graphene-based materials, their genotoxic effects, and the in vitro and in vivo assays to detect them.

GBMs, similar to other nanomaterials, could be genotoxic through primary or secondary mechanisms of action [29] (Figure 2). Primary genotoxicity could be induced by direct interaction of the material with the DNA molecule or its associated histones, or indirectly, by interfering with DNA replication or repair through the induction of lipid peroxidation, or by triggering the formation of reactive oxygen species (ROS). On the other hand, genotoxicity may be induced by a secondary mechanism, as a result of the interaction of the material with inflammatory cells that cause downstream secondary effects in the target cell [30,31]. Standard in vitro assays are typically performed in monocultures of non-inflammatory cells. Hence, they can only detect genotoxic effects induced by primary mechanisms of action. On the other hand, in vivo experiments performed in the whole organisms allow the detection of genotoxic effects induced by both primary and secondary mechanisms of action [30].

The aim of the present review is to evaluate the genotoxic and mutagenic potential of GBMs to humans based on the existing literature. We have performed a systematic review by collecting the information published in the last 10 years. We critically discuss the suitability of the assays used for assessing GBMs’ genotoxicity, outline the knowledge gaps that still exist, and provide recommendations for future genotoxicity studies with GBMs. 

## 2. Search Strategy

A bibliographic search was carried out through PubMed and Scopus databases by entering key words in the advanced search builder. These key words were: graphene AND (genotoxic* OR mutagenic*) (included in the title/abstract). A filter was applied to select articles published in the last 10 years (from 31 August 2011 to 31 August 2021).

In this first phase of the selection process, 86 articles were retrieved through the PubMed database search, and 127 articles through the Scopus database search. After deleting repeated references, the final number of articles retrieved through both databases was 143, which were assessed for eligibility.

In the first phase, the articles were selected by reading the abstracts and applying the following inclusion and exclusion criteria:

Inclusion criteria:

- The article contains information on in vitro, in vivo, in silico or human biomonitoring genotoxicity testing of GBMs.
- Exclusion criteria:
- The topic of the article is out of the scope of this review;
- Full text was not available in English through conventional sources;
- It is a review article;
- The article deals with environmental and ecotoxicity studies;
- The article deals with gene expression assessments.

In cases of doubt, the article was selected for in depth analysis in the second phase; this involved reading the full article.

After applying the inclusion and exclusion criteria, 50 articles were selected. 

## 3. Results and Discussion

### 3.1. Overview of the Literature Search

#### 3.1.1. Genotoxicity Studies and Assays

**Figure 3:**
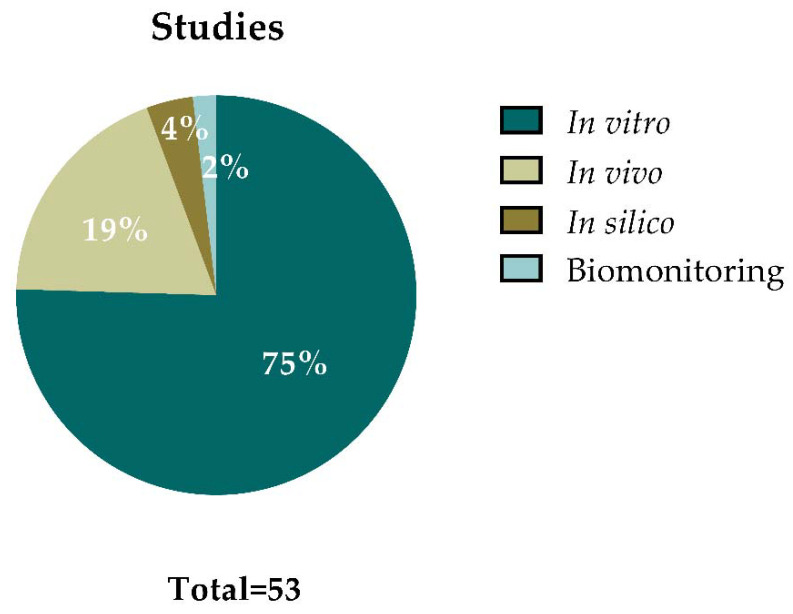
Overview of the 53 genotoxicity studies analyzed in this review. The percentage of each type of study (in vitro, in vivo, in silico, and biomonitoring studies) is indicated inside the different categories represented in the figure.

Figure 3 summarizes the outcomes from the 50 articles that were selected for data extraction. Some of the articles included different types of studies, resulting in a total of 53 studies (i.e., in vitro, in vivo, in silico, or human biomonitoring approaches). From all these studies, 40 of them (75%) were performed in vitro, 10 (19%) were carried out in vivo, 2 studies (4%) applied in silico approaches, and 1 study (2%) provided data on human biomonitoring. This demonstrates that most of the studies regarding GBMs’ genotoxic potential are focused on in vitro approaches.

**Figure 4:**
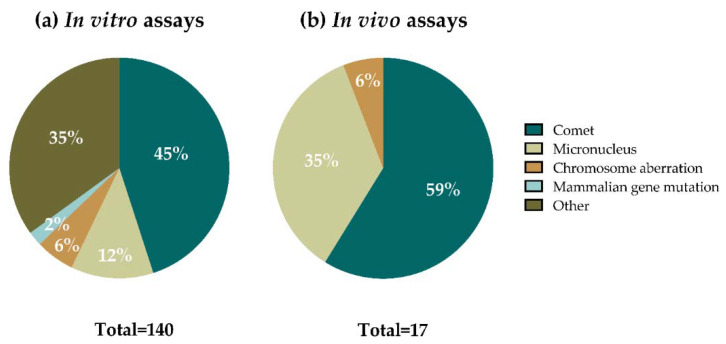
Percentages of the different assays carried out using (**a**) in vitro and (**b**) in vivo approaches among the evaluated publications.

In most of the studies, several assays (understanding assay as one material analyzed in one cell line with one method) were included. From a total of 140 in vitro assays performed (Figure 4a), 63 of them (45%) accounted for comet assay experiments in mammalian cells, using both the standard and the enzyme-modified versions of the assay, 17 (12%) used the micronucleus (MN) assay, 8 assays (6%) were performed using the chromosomal aberrations (CA) method, and 3 (2%) were mammalian gene mutation assays. The other 49 in vitro methods (35%) included the γ-H2AX assay, the Ames test, non-conventional modified comet assay versions, a non-conventional plasmid DNA mutation assay, and methods for detecting DNA fragmentation. Regarding the in vivo assays (Figure 4b), from a total of 17 assays, most of them were performed using the comet assay (10 assays, 59%) and the MN test (6 assays, 35%), and 1 assay (6%) where data on CA was provided.

Among the methods used in nanogenotoxicology, the comet assay has been the most extensively performed one [26,27,28], and GBMs, according to the results in Figure 4, are not the exception. Almost half of the in vitro assays, and more than half of the in vivo assays, were performed following the conventional comet assay protocols (standard comet and formamidopyrimidine DNA glycosylase (Fpg)-modified comet assays). In addition to that, non-conventional comet assay versions were used in two additional in vitro studies focused on graphene-based nanocomposites (NCs), as well as in one study using yeast as an experimental model (included in the “other” category).

The single-cell gel electrophoresis or comet assay detects DNA strand breaks (SBs) and alkali-labile sites at a single cell level with high sensitivity [32] (Figure 2). In this assay, cells or nuclei are embedded in agarose and lysed forming nucleoids. After an alkaline treatment followed by an electrophoresis, DNA is able to migrate if breaks are present and form comet-like images. The relative amount of DNA in the tail of the comets (often reported as % tail DNA) reflects the level of breaks. Moreover, the incubation of nucleoids with lesion-specific enzymes allows the detection of base alterations [33]. In this sense, the bacterial enzyme Fpg is widely used for the detection of oxidized bases, a lesion known to be caused by many nanomaterials, as an effect of inducing oxidative stress. Among the advantages of the comet assay are its full applicability in vitro, in vivo, and in biomonitoring studies, the potential to detect DNA damage in different tissues, the applicability to proliferating or non-proliferating cells, and the small amount of sample required. An OECD guideline [34] for the in vivo comet assay was published in 2014 and updated in 2016. As mentioned before, the in vitro version has not yet been validated.

Critical points of this assay that can influence the % of DNA in tail have been identified (e.g., agarose concentration and electrophoresis conditions) and several recommendations to perform both the in vitro and the in vivo versions have been published [35]. Data of cell proliferation and colony forming efficiency (CFE) have been applied as a cytotoxicity measurement in nanomaterial testing [36,37,38]; it seems that a cut-off of 20% reduced proliferation or CFE could be used to discriminate potential artifacts. Nevertheless, nanomaterials or their agglomerates can interfere with toxicity tests, interacting with non-nucleosomal DNA and/or inducing DNA damage during the processing steps [37,39]. They also can complicate the analysis due to overlapping of nanomaterials and comets. In general, the lack of standardized methods and the potential interference with GBMs should be critically evaluated in order to allow proper interpretation of the results.

As abovementioned, DNA lesions detected by the comet assay may be reparable by the DNA repair machinery present in all cells. However, if they are not repaired, or misrepaired, when the cell replicates they can be the basis for mutations. Due to this fact, these DNA lesions are also called pre-mutagenic lesions.

The MN assay is the second most used approach in the assessment of GBMs’ genotoxicity (Figure 4), as well as of other nanomaterial [16]. The test is based on the detection of chromosome fragments (clastogenic effect) or whole chromosomes (aneugenic effect) in the form of micronuclei, which were formed during the previous cell division [40]. Opposite to the comet assay, the damage detected by the MN assay is not repairable (Figure 2). Both the in vitro and the in vivo versions of the assay have validated OECD guidelines (Test Guideline (TG) 474 and TG 487, respectively) [41,42]. The cytochalasin-blocked micronucleus (CBMN) assay is the most used approach and includes the use of cytochalasin B (cyt-B), a cytokinesis blocking agent which allows for restricting the analysis of micronuclei in binucleated cells [40]. However, cyt-B may impair nanomaterial intracellular uptake, leading to false negative results [43,44]. Therefore, the addition of cyt-B to cell cultures after treatment must be delayed when testing nanomaterials [23]. Nowadays, automated analysis systems (e.g., flow cytometry) facilitate the scoring of micronuclei, but interferences between nanomaterials and MN could arise [45]. In the case of the in vivo MN test, the assessment is done in bone marrow or peripheral blood erythrocytes. Hence, the assay may not be suitable for nanomaterials if they accumulate in organs other than the hematopoietic system or require longer exposure schemes than those recommended by the guidelines to reach the target organ, which is the bone marrow [46]. 

The CA test was mainly used in in vitro studies (eight assays), and in one in vivo study, representing the third most applied method to reveal GBMs’ genotoxicity (Figure 4). This test intends to identify agents that induce structural CA both in cultured mammalian and in bone marrow cells of animals, as described in OECD TG 473 and 475 for the in vitro and in vivo approaches, respectively [47,48]. However, due to the high level of expertise and time required for the analysis, this assay is much less used than the MN assay [26]. 

Despite being a validated method [49], only three assays were performed with the in vitro mammalian gene mutation method (Figure 4). Two different cell lines can be used for this assay: L5178Y TK^+/−^ −3.7.2 C mouse lymphoma and TK6 human lymphoblastoid cell lines. The exposure of these cells to a test agent may induce non-repairable gene mutations or chromosomal events (namely, loss of heterozygosity, LOH) turning the autosomal and heterozygous TK^+/−^ gene into TK^−/−^ (Figure 2). The major limitation of the method is that conditions such as pH, and osmolarity can easily lead to false-positive results [49]. However, due to the limitations of other tests to evaluate nanomaterials’ potential to induce gene mutations (e.g., Ames test), mammalian gene mutation assays are mandatory under the regulatory umbrella [23,50]. Nevertheless, the limited number of studies on nanomaterials with these assays, together with the absence of parallel in vivo data, precludes the evaluation of the reliability of this assay in detecting mutagenic nanomaterials [16].

Among the other assays used to study genotoxicity in vitro, six were performed with the γ-H2AX assay, which is widely used as a marker of double-strand DNA breaks (DSBs) [51]. Although not being validated by regulatory administrations, the method predictability, specificity, and sensitivity have been proven in numerous studies [52]. The main limitation when applying this method to nanomaterials is that nanomaterials may mask the γH2AX signal labeling [53]. 

The rest of the assays were carried out with the following tests: Ames test (six assays), DNA fragmentation assays, mostly after cellular exposure to GBMs (twenty-six assays), but also after the naked DNA exposure to GBMs (four assays), the above mentioned non-conventional comet versions (six assays), and one gene mutation assay using plasmid DNA (Figure 4).

#### 3.1.2. Evaluated GBMs

**Figure 5:**
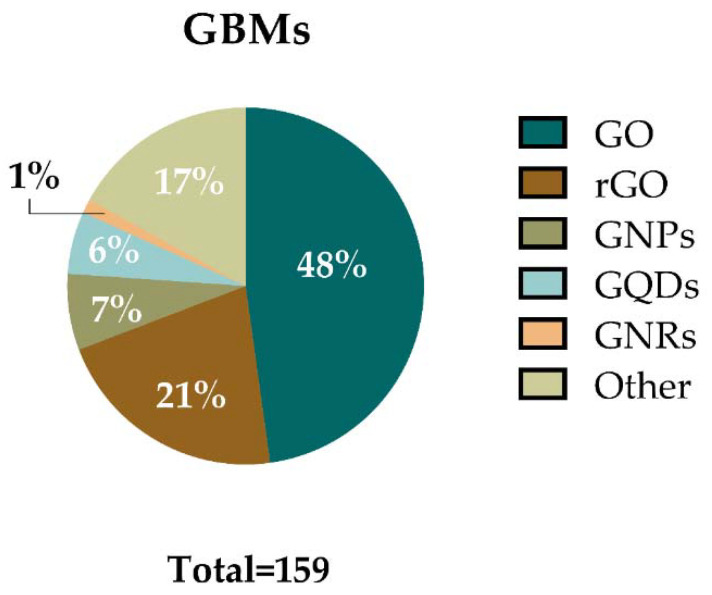
Percentages of the in vitro, in vivo and biomonitoring assays carried out using different GBMs (graphene oxide, GO; reduced GO, rGO; graphene nanoplatelets, GNPs; graphene quantum dots, GQDs; graphene nanoribbons, GNRs; and others).

Regarding the GBMs used in the assays analyzed, the materials have been classified in six main categories: GO, rGO, graphene quantum dots (GQDs), graphene nanoplatelets (GNPs), graphene nanoribbons (GNRs), and “other GBMs”. The latter includes FLG, functionalized FLG, and exfoliated graphene among others. Figure 5 shows the percentages of assays in which these materials were used. Each category includes the main material and a variety of formulas related to that material (e.g., GO category includes GO, different functionalized GOs, and different layered GO such as nanosheets, or few-layer GO). In agreement with the recent review of Achawi and colleagues [5], GO was the most studied material. GO or GO-based materials were employed in 76 assays (48%) included in this review. It was followed by rGO and its related materials, e.g., reduced GO nanoplatelets (rGONPs), reduced GO nanoribbons, (rGONRs), few-layer rGO, and different types of functionalization which were used in 34 assays (21%). GNPs and GNP-based materials were used in 11 assays (7%), while 9 assays (6%) were performed with GQDs and their derivates. Only two assays (1%) used GNRs. Other GBMs were used in 27 assays (17%).

The main PC parameters measured in the reviewed articles were thickness, lateral size, zeta potential, and hydrodynamic diameter. However, although not so commonly, other parameters such as surface area [54,55,56], chemical composition [56,57,58,59,60,61,62,63,64,65,66], impurity concentration [56,57,63,64,67,68], aggregation/agglomeration [54,60,63,64,69,70], morphology and/or surface characteristics [55,57,71,72], thermal stability [67], elasticity [56], precipitation in suspension [65], number of layers [54,57,60,61,63,64,71,73,74,75,76,77] hydrophobicity [70], and electrical conductivity [56], among others, were also determined in some of the studies. The lack of a comprehensive PC report of the GBMs observed in most of the reviewed publications makes it difficult to compare the genotoxic effects reported in the different studies.

The key PC parameters that should be included in studies reporting the biological effects of GBMs include dimensions (average lateral size, diameter, or agglomeration status), number of layers, oxidation state, and presence of impurities [2,4,5]. In the case of functionalized materials, data on the surface functional groups should also be reported.

### 3.2. In Vitro Studies

Among the selected articles, only one of them [58] tested the same material with three validated in vitro assays (MN, CA, and gene mutations). Two other articles combined the comet and the CA assays for the same materials [73,74]. The rest of the in vitro studies mainly used only one type of assay when testing the same material. Therefore, the results of the studies are reported below separately for each assay.

#### 3.2.1. Chromosome Damage Assays

Table 1 and Table 2 summarize the in vitro studies that were selected in the literature search performed using methods that detect non-repairable chromosome damage, regardless of whether the authors follow or not established OECD guidelines. Nevertheless, we have followed the guidelines’ criteria on cytotoxicity for discussing the outcomes of the genotoxicity evaluations. For MN and CA, as stated in the guidelines, the highest tested dose should not exceed 55 ± 5% cytotoxicity measured by the recommended methods [42,47]. On the other hand, cellular uptake, which should be assessed to substantiate negative outcomes [23], has also been considered in our discussions.

A total of six publications analyzed MN formation after exposure to GBMs [58,63,64,65,66,78] (Table 1). Most of these publications used the conventional CBMN assay for this purpose, but a flow cytometer-based method using the MicroFlow Kit (Litron, Rochester, NY) was also performed in the study by Petibone et al., 2017 [58]. Only in three studies did the authors follow the guidelines established by the OECD [58,64,65]. Cell proliferation is considered a relevant piece of information in this assay since MN formation requires cell division during or after the exposure to the test agent. Furthermore, concurrent cytotoxicity analyses should be performed using cell proliferation-based parameters. 

Regarding the material studied, GO, graphene platelets, and different functionalization of FLG were investigated. Three studies tested different forms of GO on lymphocyte cell lines (Jurkat, WIL2-NS) and primary lymphocytes [65], human fibroblasts [78], and L929 and MCF-7 cell lines [66]. Only GO nanosheets significantly increased MN frequency in human primary lymphocytes after 44 h of exposure at 50 and 100 µg/mL [65]. On the other hand, another type of GO (10 µg/mL) did not induce MN formation in human fibroblasts after 24 h of exposure [78]. Similarly, GO-Fe_3_O_4_ (3.125–100 µg/mL) did not increase MN frequency in L929 and MCF-7 cell lines after a 48-h exposure [66]. 

In the study carried out by Petibone et al. [58], where two human lymphoblastoid cell lines (TK6 and NH32) were exposed to oxygen functionalized graphene platelets (1–100 µg/mL) for 4 h, they reported neither an increase in MN nor hypodiploid cells frequencies in any of the cell lines, even though cellular uptake was confirmed. However, other indicators of genotoxicity, such as the frequency of aberrant cells in NH32 cells and the increase of TK^+/-^ LOH frequency, showed significant values after being exposed to platelets (50 and 100 µg/mL, 4 h) in both cell lines (Table 2 and Table 3). Nevertheless, increased LOH frequencies in TK6 cells should be consider with caution as this cell line showed cytotoxicity in the same conditions.

**Table 2:** In vitro studies assessing the genotoxicity of GBMs using the chromosome aberration assay.

Material	Characterization ^a^	Cell Line	Dose Range (µg/mL) and Treatment Time	Result ^b^	Cytotoxicity ^c^	Reference
Bacterial reduced GO	NA	PBMNCs	100, 600 μg (24 h)	Negative	Negative	[79]
	1.7 nm					
	NA					
	NA					
rGONPs	11 ± 4 nm	hMSCs	0.01–100 (1 h)	Positive (0.1)	Positive (100)	[73]
	1.1–2.3 nm					
	NA					
	NA					
	91 ± 37 nm			Positive (1)	Negative	
	1.1–2.3 nm					
	NA					
	NA					
	418 ± 56 nm			Negative		
	1.1–2.3 nm					
	NA					
	NA					
	3.8 ± 0.4 μm			Negative		
	0.7 nm					
	NA					
	NA					
rGONRs	10 μm	hMSCs	0.01–100 (1 h)	Positive (1)	Negative	[74]
	1 nm		0.01–100 (5 h)	Positive (0.1)		
	NA		0.01–100 (24 h)		Positive (100)	
	NA		0.01–100 (96 h)	Positive (0.01)	Positive (10)	
Oxygen functionalized graphene	<10 μm	TK6	10–100 µg/mL (4 h)	Negative ^d,e^	Negative	[58]
	1–1.2 nm					
	−64.66 ± 9.83 mV	NH32		Positive (100)		
	237.1 ± 17.2 nm					

^a^ Characterization of GBM is indicated as follows: lateral size, thickness, Z-potential, hydrodynamic diameter. NA: information not available. ^b^ The lowest dose (µg/mL) at which positive results are reported is indicated in brackets. ^c^ The evaluation of cytotoxicity has resulted in a reinterpretation of the authors’ results, based on the recommended 55 ± 5% cytotoxicity limit established in the OECD TG 473 [47] for non-toxic concentrations. Every cytotoxicity assay has been considered, regardless of whether it was not a proliferation assay recommended by the OECD TG 473 [47]; the lowest dose (µg/mL) at which positive results are reported is indicated in brackets. ^d^ Cellular uptake was assessed. ^e^ Cellular uptake was confirmed. PBMCs: peripheral blood mononuclear cells. hMSCs: human mesenchymal stem cells.

Burgum and co-workers assessed the induction of MN in the human bronchial epithelial 16HBE14o- cell line [63], in TT1 alveolar epithelial cell monocultures, and in TT1 and differentiated THP-1 monocytes co-cultures [64]. The in vitro models were exposed to the same neutral, aminated (NH_2_-), and carboxylated (COOH-) FLG (2–100 µg/mL) for 24 h. Neutral and aminated FLG induced MN formation in 16HBE14o- cells [63], while all forms of FLG induced MN in TT1 monocultures and TT1/THP-1 co-cultures [64]. Further experiments were carried out by Burgum et al. [63] to determine the origin (clastogenicity or aneugenicity) of the detected MN in 16HBE14o- cells using fluorescent in situ hybridization (FISH) to stain the centromeres. Neutral and aminated FLG showed the potential to induce both chromosome breaks and loss of entire chromosomes, but only amine-FLG generated significant clastogenicity. Carboxylated FLG did not induce MN in16HBE14o- cells, but cellular uptake was not assessed.

Altogether, functionalized FLG appeared to have greater potential to induce formation of MN in different cell types than GO, since MN induced using the latter were only found in one study performed with GO nanosheets in human lymphocytes [65], where the rest of studies reporting negative outcomes with GO did not confirm cellular uptake. Nevertheless, variables between studies (i.e., different cell lines and culture conditions) hamper the comparison between GBMs’ genotoxic potential.

Table 2 summarizes the results of the four publications that studied changes in chromosomal structure to determine the genotoxic potential of GBMs. The study of Giemsa-stained metaphase cells is the most frequently used method to assess this type of alteration in different cell lines after exposure to GBMs [73,74,79]. All these studies were performed using different forms of rGO-based materials, such as rGONPs [73], rGONRs [74], and bacterial rGO [79]. In addition, Petibone and colleagues [58] applied the whole chromosome FISH technique to evaluate the effects of oxygen functionalized graphene platelets.

Akhavan and colleagues used human mesenchymal stem cells (hMSCs) to carry out the CA analysis after treatment with different sizes of rGONPs [73] and rGONRs [74]. Although they did not classify the types of structural aberrations analyzed, as recommended by the OECD TG 473 [47], the results showed a significantly increased frequency of the total CA even before the appearance of cytotoxic effects. A time-dependent genotoxic effect was reported after treatment with 0.01–100 µg/mL of rGONRs at 1, 5, 24, and 94 h [74]. Longer exposures led to genotoxic effects at lower doses. Size-dependency was reported after the treatment with rGONPs (from 11 nm to 3.8 µm) as smaller lateral size GO-based materials were more prone to cause chromosomal aberrations at lower concentrations [73]. Similar results were obtained to the comet assay, in which the data followed the same trend at non-cytotoxic concentrations [73,74]. On the other hand, the ability of another rGO material (bacterial rGO) to induce chromosome and chromatid breaks and gaps, or other types of aberrations in peripheral blood mononuclear cells (PBMCs), was studied by Cherian et al. [79]. The authors did not find a significant increase in the frequency of the analyzed CA, although cellular internalization was not assessed.

The FISH method was used to detect chromosomal aberrations in the study performed by Petibone et al. [58]. The authors evaluated the frequency of total aberrant cells, cells with color junctions, cells with translocated chromosomes, and cells with dicentrics or acentrics after the exposure of TK6 or NH32 cells to oxygen functionalized graphene platelets (10–100 μg/mL, 4 h). Results demonstrated the lack of effects in the TK6 cell line, while the frequency of total NH32 aberrant cells and acentrics was significantly increased after exposure to a “moderate-cytotoxic” (as stated by the authors) concentration of platelets (100 μg/mL). In addition, the authors reported significant changes in the frequencies of NH32 cells with color junctions, translocated chromosomes, and dicentrics. However, the authors also reported that the exposure to the graphene platelets induced cell cycle arrest and apoptosis in TK6 cells; therefore, the results obtained with this cell line should be carefully considered.

#### 3.2.2. Gene Mutation Assays

The results of the gene mutation assays, which are able to detect unrepairable damage, are shown in Table 3. The validated TK^+/−^ mutation assay was performed by Petibone et al. [58] using TK6 and NH32 cells. The *TK* LOH frequency increased significantly after the exposure to 50 and 100 µg/mL, and to 1, 50, and 100 µg/mL of the graphene material in TK6 and NH32 cell lines, respectively. Demir and Marcos [80] evaluated the same target (*TK* gene) using L5178Y/TK^+/−^−3.7.2 C mouse lymphoma cells. The authors reported the lack of effects after the treatment with graphene nanoplatelets for 4 h, although cellular uptake was not assessed.

**Table 3:** In vitro studies assessing the genotoxicity of GBMs using the mammalian gene mutation assay.

Material	Characterization ^a^	Cell Line	Dose Range (µg/mL) and Treatment Time	Result ^b^	Cytotoxicity ^c^	Reference
GNPs	NA	L5178Y/Tk^+/−^−3.7.2 C	0.01–250 (4 h)	Negative	Negative	[80]
	2–18 nm					
	−7.68 ± 0.45 mV					
	117.8 ± 4.12 nm					
Oxygen functionalized graphene	<10 μm	TK6	1–100 (4 h)	Positive (1, 50, 100)	Negative	[58]
	1–1.2 nm					
	−64.66 ± 9.83 mV	NH32				
	237.1 ± 17.2 nm					

^a^ Characterization of GBM is indicated as follows: lateral size, thickness, Z-potential, hydrodynamic diameter. NA: information not available. ^b^ The doses (µg/mL) at which positive results are reported are indicated in brackets. ^c^ The evaluation of cytotoxicity has resulted in a reinterpretation of the authors’ results, based on the recommended 20–10% viability limit established in the OECD TG 490 [49] for non-toxic concentrations. Every cytotoxicity assay has been considered, regardless of whether it was or was not a proliferation assay recommended by the OECD TG 490 [49]; the lowest dose (µg/mL) at which positive results are reported is indicated in brackets. Cellular uptake was not assessed in the study reporting negative results.

#### 3.2.3. Assays Detecting Premutagenic DNA Damage

Twenty-four of the articles included the evaluation of the induction of premutagenic DNA damage by the in vitro comet assay (Table 4, Table 5 and Table 6). As mentioned above, the comet assay was the most used one among the in vitro evaluations. In all the studies, the standard version of the comet assay was conducted. Only in one of them was the Fpg-modified version also carried out [70]. For the comet assay, testing of non-toxic concentrations based on cell proliferation assays (relative suspension growth > 80%) is highly advised [36,37,38], as the use of excessively toxic concentrations could lead to false positive results [16]. This criterion was applied for the evaluation of cytotoxicity with the aim to discuss the outcomes of the genotoxicity assessments. In all the reviewed studies, a cytotoxicity evaluation was carried out using several methods (i.e., MTT, fluorescence diacetate assay, trypan blue, ATP or NAD+/NADH production or evaluation of membrane integrity). However, none of them included cell proliferation assays to set a relevant concentration range for the genotoxicity analysis. Furthermore, none of the studies reporting negative results, except for Domenech and colleagues [70] and Lin and colleagues [61] confirmed the internalization of the materials into the cells. Hence, the negative results should be interpreted with caution. 

**Table 4:** In vitro studies assessing the genotoxicity of graphene oxide (GO) and GO-related materials using the comet assay.

Material	Characterization ^a^	Cell Line	Dose Range (µg/mL) and Treatment Time	Result ^b^	Cytotoxicity ^c^	Reference
GO	NA	U87	50 (24 h)	Positive (50)	Negative	[55]
	NA					
	−9.6 mV					
	NA					
GO	NA	Colon 26	1–50 (24 h)	Negative	Positive (0.1)	[62]
	1–2, 3–4 nm					
	−24.5 ± 0.4 mV					
	250 ± 68 nm, 1.5 ± 7 µm					
GO	NA	ARPE-19	100 (24 h)	Positive (100)	Negative	[68]
	1.4 ± 0.2 nm					
	NA					
	NA					
GO	1065.8 ± 251.5 nm	A549	100 (24 h)	Positive ^e,f^ (100)	Positive (100)	[81]
	NA					
	−48.6 ± 2.4 mV					
	1944 ± 89.1 nm					
GO	0.8 nm	SSCs	1–400 (24 h)	Positive (10)	Positive (100)	[57]
	NA					
	NA					
	NA					
GO	NA	Spermatozoa	0.1–400 (2 h)	Positive (100)	Positive (1)	[82]
	NA					
	−41.2 ± 3.1 mV					
	NA					
GO	NA	Caco-2/HT29 barrier	5–50 (24 h)	Positive (5) Negative ^d,e^	Negative	[70]
	89.42 ± 8.30 nm					
	−15.6 ± 0.4 mV					
	244.9 ± 7.4 nm					
GO	NA	HepG2	4–50 (24 h)	Negative	Positive (25)	[72]
	NA					
	−33.7 ± 0.4 mV					
	280 nm–6.4 µm					
GO	200–500 nm	HLF	1–100 (24 h)	Positive (1)	Positive (50)	[83]
	1 nm					
	−65.1 mV					
	NA					
GO	1.32 µm	A549 Caco-2 Vero	10–100 (24 h)	Positive (50)	Negative	[77]
	NA					
	NA					
	NA					
GO	130 nm	A549 Caco-2 Vero	10–100 (24 h)	Positive (50)	Negative	[77]
	NA					
	NA					
	NA					
GO	300–800 nm	H9c2	10–100 (24 h)	Positive (40)	Positive (20)	[67]
	0.7–1.2 nm					
	NA					
	NA					
GO	100 ± 50 nm	BEAS-2B	12.5–25 (24 h)	Negative	Negative	[76]
	NA					
	NA					
	NA					
GO	10 ± 8 µm	BEAS-2B	12.5–25 (24 h)	Negative	Negative	[76]
	NA					
	NA					
	NA					
GO	10 µm	BEAS-2B	10–50 (NA)	Positive (50)	Negative	[75]
	21 nm					
	8.98 ± 0.55 mV					
	NA					
MPO-degraded GO	100 ± 50 nm	BEAS-2B	12.5–25 (24 h)	Negative	Negative	[76]
	NA					
	NA					
	NA					
MPO-degraded GO	10 ± 8 µm	BEAS-2B	12.5–25 (24 h)	Negative	Negative	[76]
	NA					
	NA					
	NA					
Few-layered GO	2–3 µm/1 µm	FE1	5–200 (24 h)	Negative	Negative	[54]
	NA					
	−39.9 ± 1.5 mV					
	157 nm					
Few-layered GO	10 µm	BEAS-2B	10–50 (NA)	Positive (50)	Negative	[75]
	122 nm					
	−9.33 ± 0.45 mV					
	NA					
PEI-GO	200–500 nm	HLF	1–100 (24 h)	Positive (50)	NA	[83]
	2.5 nm					
	60.5 mV					
	NA					
PEG-GO	50–150 nm	HLF	1–100 (24 h)	Positive (100)	NA	[83]
	1.9 nm					
	−8.86 mV					
	NA					
LA-PEG-GO	100–200 nm	HLF	1–100 (24 h)	Negative	NA	[83]
	2 nm					
	18.4 mV					
	NA					
GO-NH_2_	NA	Colon 26	1–50 (24 h)	Negative	Positive (1)	[62]
	1–2, 3–4 nm					
	38.5 ± 2.8 mV					
	560 ± 300 nm					
haGO-NH_2_	594 ± 270 nm	HepG2	4–50 (24 h)	Negative	Positive (4)	[72]
	NA					
	−12.28 ± 0.6 mV					
	NA					

^a^ Characterization of GBM is indicated as follows: lateral size, thickness, Z-potential, hydrodynamic diameter. NA: information not available; MPO: human mieloperoxidase; PEI: polyethylenemine; PEG: poly(ethylene glycol); LA-PEG: lactobionic acid poly(ethylene glycol); ha: hydroxylamine. ^b^ The lowest dose (µg/mL) at which positive results were reported is indicated in brackets. ^c^ The evaluation of cytotoxicity has consisted in a reinterpretation of the authors’ results, based on the recommended 80% cell viability for non-toxic concentrations. Every cytotoxicity assay has been taken into account, regardless of whether it was or was not a proliferation assay; the lowest dose (µg/mL) at which positive results are reported is indicated in brackets. ^d^ Material evaluated by the Fpg-modified version of the comet assay. ^e^ Cellular uptake was assessed. ^f^ Cellular uptake was confirmed. SSCs: spermatogonial stem cells; HLF: human lung fibroblast cells.

A large number of different cell lines were used for the evaluations. Regarding the evaluated GBMs, a wide variety of them were assessed with this assay, including single and few-layered GO and rGO, functionalized GO and rGO, rGONPs, rGONRs, GNPs, functionalized GNPs, GQDs, functionalized GQDs, pristine graphene, and functionalized graphene.

Two of the articles selected did not provide enough data of the concentrations tested [56,84]. Furthermore, the information provided by Senel and colleagues [84] was not enough to correctly understand the results obtained.

The results of the GO evaluations are shown in Table 4. GO reported a majority of positive results (18 out of 28 assays). However, it should be noted that in some of the articles, positive responses were observed at highly cytotoxic concentrations [67,82]. Furthermore, Wang and colleagues [83] carried out the cytotoxicity assays only with GO to set the concentrations of the functionalized GO tested. In the case of Rozhina and colleagues [81], only one GO concentration, which showed a 20% apoptosis in the cytotoxicity assay, was tested and reported a positive result. As cellular internalization was quite low, negative outcomes at lower doses are uncertain. Wang and colleagues [83] observed a reduction in the genotoxic effect of GO in human lung fibroblast (HLF) cells after being functionalized with different surface groups. Unfunctionalized GO showed the strongest genotoxic effects, followed by polyethylenimine functionalized GO (PEI-GO), and polyethylene glycol functionalized GO (PEG-GO); whereas lactobionic acid-polyethylene glycol functionalized GO (LA-PEG-GO) did not show genotoxic effects at the concentrations tested (1–100 µg/mL). Positive results were obtained with the human broncho epithelial BEAS 2B cell line treated with different GO only when concentrations of 50 µg/mL or higher were included [75]. A similar behavior was observed with another human bronchial A549 cell line [77,81], except when treated with lower concentrations (10 µg/mL) of a 130 nm GO [77]. On the other hand, negative results were observed after treating a mouse lung epithelial FE1 cell line with up to 200 µg/mL of few-layered GO [54]. Regarding other cell lines, positive results with concentrations below 50 µg/mL were reported for spermatogonial stem cells (SSCs) [57], Caco-2/HT29 barrier [70], HLF [83], A549, human colorectal adenocarcinoma Caco-2, monkey kidney epithelial Vero [77] and embryonic rat heart H9c2 cells [67]. The latter at high cytotoxic doses and without an assessment of cellular uptake. Negative results were reported when using the human hepatoma HepG2 cell line [72]. Nevertheless, the incomplete PC characterization of many analyzed materials together with the wide range of cell types used in the tests does not allow for the identification of properties that may affect the genotoxic results.

As previously mentioned, DNA damage induced by oxidative stress is a well-established mechanism of action of GBMs [29]; however, GO (and also GNPs) reported positive results in the standard comet assay but negative ones in the Fpg-modified comet assay [70].

The results of the rGO evaluations using the comet assay are shown in Table 5. In all the studies reporting positive outcomes, the genotoxic effects were obtained with too cytotoxic doses, compromising the interpretation of these results. Only two exceptions were found. In the study of Akhavan et al. [73], they found a significant induction of DNA damage after 1 h exposure of human mesenchymal stem cells (hMSCs) to 0.1 and 1 µg/mL of 11 nm and 91 nm rGONPs, respectively. A positive response was also observed at 100 µg/mL for 418 nm and 3.8 µm rGONPs in the same study, but these concentrations were too cytotoxic. As internalization of the materials was not confirmed, the negative results reported at lower doses should be considered with caution. A year later, the same authors [74] observed a significant induction of DNA damage in hMSCs after exposure to 1 µg/mL rGONRs for 1 and 5 h, and to 0.1 µg/mL rGONRs for 24 and 96 h. One study reported negative results in spermatozoa treated with green tea polyphenols-rGO (GTP-rGO) despite testing a wide range of concentrations up to 400 µg/mL [82]. Positive responses were obtained for NH2H4-rGO and hydrothermal-rGO (HT-rGO) in the same study [82], but at concentrations clearly cytotoxic. No cellular uptake was assessed in this case. On the other hand, few-layered rGO evaluated by Bengston and colleagues [54] reported negative results after exposing FE1 cells to 5–200 µg/mL for 24 or 34 h. 

**Table 5:** In vitro studies assessing the genotoxicity of reduced graphene oxide (rGO) and rGO-related materials rGOs using the comet assay.

Material	Characterization ^a^	Cell Line	Dose Range (µg/mL) and Treatment Time	Result ^b^	Cytotoxicity ^c^	Reference
rGO	NA	U87	50 (24 h)	Positive ^d,e^ (50)	Positive (50)	[55]
	NA					
	−38.3 mV					
	NA					
rGO	0.8 nm	SSCs	1–400 (24 h)	Negative	Positive (100)	[57]
	NA					
	NA					
	NA					
rGO-3	NA	ArRPE-19	100 (24 h)	Positive (100)	Positive (100)	[68]
rGO-6	1.4 ± 0.2 nm					
rGO-9	NA					
rGO-12	NA					
Few-layered rGO	1–2 µm/0.5–2 µm	FE1	5–200 (3 h)	Negative	Negative	[54]
	NA					
	−10.7 ± 0.6 mV		5–200 (24 h)			
	274 nm					
Few-layered rGO	1–2 µm/0.2–8 µm	FE1	5–200 (3 h)	Negative	Negative	[54]
	NA					
	−12.2 ± 0.6 mV		5–200 (24 h)			
	284 nm					
rGONPs	11 ± 4 nm	hMSCs	0.01–100 (1 h)	Positive (0.1)	Positive (10)	[73]
	1.1–2.3 nm					
	NA					
	NA					
	91 ± 37 nm			Positive (1)	Positive (10)	
	1.1–2.3 nm					
	NA					
	NA					
	418 ± 56 nm			Positive (100)	Positive (100)	
	1.1–2.3 nm					
	NA					
	NA					
	3.8 ± 0.4 µm			Positive (100)	Positive (100)	
	0.7 nm					
	NA					
	NA					
rGONRs	10 μm	hMSCs	0.01–100 (1 h)	Positive (1)	Positive (100)	[74]
	1 nm		0.01–100 (5 h)	Positive (1)	Positive (10)	
	NA		0.01–100 (24 h)	Positive (0.1)	Positive (10)	
	NA		0.01–100 (96 h)	Positive (0.1)	Positive (1)	
rGOM	NA	RPE	NA (48 h)	Negative	NA	[56]
		PHCLC				
		PHCK				
		Arpe-19				
		HCE-T				
NH_2_H_4_-rGO	NA	Spermatozoa	0.1–400 (2 h)	Positive (100)	Positive (0.1)	[82]
	NA					
	−20.9 ± 1.7 mV					
	NA					
HT-rGO	NA	Spermatozoa	0.1–400 (2 h)	Positive (100)	Positive (0.1)	[82]
	NA					
	−36.4 ± 2.1 mV					
	NA					
GTP-rGO	NA	Spermatozoa	0.1–400 (2 h)	Negative	Positive (0.1)	[82]
	NA					
	−27.1 ± 2.5 mV					
	NA					

^a^ Characterization of GBM is indicated as follows: lateral size, thickness, Z-potential, hydrodynamic diameter. NA: information not available. NH_2_H_4_: hydrazine; HT: hydrothermal; GTP: green tea polyphenols. ^b^ The lowest dose (µg/mL) at which positive results were reported is indicated in brackets. ^c^ The evaluation of cytotoxicity has resulted in a reinterpretation of the authors’ results, based on the recommended 80% cell viability for non-toxic concentrations. Every cytotoxicity assay has been taken into account, regardless of whether it was or was not a proliferation assay; the lowest dose (µg/mL) at which positive results are reported is indicated in brackets. ^d^ Cellular uptake was assessed. ^e^ Cellular uptake was confirmed. SSCs: spermatogonial stem cells; hMSCs: human mesenchymal stem cells; RPE: retinal pigment epithelium cells; PHCLC: primary human corneal limbal cells; PHCK: primary human corneal keratocytes.

Table 6 summarizes the results obtained with other GBMs using the comet assay. GNPs showed a significant induction of DNA damage in a Caco-2/HT29 co-culture system mimicking the intestinal barrier at concentrations as low as 5 µg/mL when using the alkali comet assay [70]. However, the results were negative when using the Fpg-modified version of the assay. Chaterjee and colleagues [75] compared unfunctionalized GNPs with those functionalized with carboxyl and amine groups in human bronchial epithelial BEAS-2B cells. All the materials had already induced positive results at the lowest tested concentration (10 µg/mL). GQDs [84] and pristine graphene (GN) [55] also reported positive outcomes, but at overly cytotoxic doses, and assessed cellular internalization. Finally, negative results were reported with G-OH in a human retinal pigment epithelial (ARPE-19) cell line [61], and with GN in peripheral blood mononuclear cells [71], although the latter were only treated for a short time (1 h).

Some interesting findings were observed in the studies. In two of the articles, a size-dependent effect was observed, showing particles with smaller lateral size having the strongest genotoxic effect [73,77]. Ou and colleagues [68] observed that GO induced lower levels of DNA damage than rGO, although at high cytotoxic doses. The authors suggested that oxygen-containing functional groups play an essential role, with saturated C-O bonds reducing genotoxicity in comparison with unsaturated C=O bonds. 

In addition to the above reported studies, the potential of GBMs to induce DNA damage was also assessed using other approaches. Two studies considered the expression of the γ-H2AX protein by immunostaining [78] and by Western blot [75]. Chatarjee and colleagues [75] used this method to detect DSBs in BEAS-2B cells exposed to 10 mg/L GNP, GNP-COOH, GNP-NH2, few-layer graphene oxide, and single-layer graphene oxide. They found that GNP-based materials induced both higher DNA damage (evaluated by the comet assay) and DSBs (evaluated by γ-H2AX protein expression) than GO-based materials. Contrarily, the exposure of human fibroblast to 10 µg/mL of GO did not lead to increased levels of the γ-H2AX protein [78].

An attempt to evaluate the ability of commercial GO and GO nanocolloids to induce genotoxicity in the yeast *Saccharomyces cerevisiae* was conducted by using the comet assay [85]. However, the authors concluded that the approach was not suitable for the determination of GBMs’ genotoxicity in yeast due to interference with the analyses. Results of DNA diffusion around the nucleus were reported by Qiao et al. [78] using the HaloChip assay. After being exposed to GO, human fibroblasts were embedded in an agarose gel in alkaline conditions and genotoxicity was observed at 100 and 500 µg/mL after 24 h treatment. However, the detected damage seems not to turn into permanent damage, since no induction of MN was detected in the same study.

Owing to the huge range of applications of GBMs, they have been used to synthesize NCs. Graphene-based NCs have been exploited as scaffolds for the immobilization of different enzymes or to develop biosensors. Specifically, Gr@Fe_3_O_4_ and Ag@rGO NCs were used by Khan et al. [86] and Shafi et al. [87], respectively, to immobilize β-galactosidase. The genotoxic potential of these NCs was assessed in isolated human lymphocytes using a modified comet assay protocol where the lymphocytes are exposed to graphene-based NCs after being immobilized in agarose. The subsequent steps follow the general procedure. Lymphocytes showed no significant levels of DNA damage after the treatment with both NCs. 

**Table 6:** In vitro studies assessing the genotoxicity of GNPs, GQDs, G, GFC, and GN using the comet assay.

Material	Characterization ^a^	Cell Line	Dose Range (µg/mL) and Treatment Time	Result ^b^	Cytotoxicity ^c^	Reference
GNPs	NA	Caco-2/HT29 barrier	5–50 (24 h)	Positive (5) Negative ^d,e,f^	Negative	[70]
	220.26 ± 33.68 nm					
	−13 ± 0.5 mV					
	243.4 ± 1.4 nm					
GNPs	10 μm	BEAS-2B	10–50 (NA)	Positive (10)	Negative	[75]
	877.2 nm					
	−14.28 ± 0.66 mV					
	NA					
GNPs-COOH	10 μm	BEAS-2B	10–50 (NA)	Positive (10)	Negative	[75]
	735.9 nm					
	−9.86 ± 0.7 mV					
	NA					
GNPs-NH_2_	10 μm	BEAS-2B	10–50 (NA)	Positive (10)	Negative	[75]
	945.5 nm					
	−10.55 ± 1.21 mV					
	NA					
N-doped GQDs	NA	NIH3T3	50–150 (24 h) (48 h)	Positive ^e,f^ (100)	Positive (15)	[84]
	NA	A549				
	−2.86 ± 1.8 mV	MDA-MB-231				
	10.9 ± 1.3 nm					
siRNA/Eu-GQDs	NA	A549	NA (72 h)	NA	NA	[84]
	NA					
	35.2 ± 1.3 mV					
	198.4 ± 4.2 nm					
G-OH	NA	ArRPE-19	5100 (24 h) (48 h) (72 h)	Negative ^e,f^	Negative	[61]
	1.3 nm					
	NA					
	NA					
GFC	NA	PBMC	6.25–25 (1 h)	Negative	Negative	[71]
	NA					
	NA					
	560–110 nm					
GN	NA	U87	50 (24 h)	Positive ^e,f^ (50)	Positive (50)	[55]
	NA					
	−9.6 mV					
	NA					

^a^ Characterization of GBM is indicated as follows: lateral size, thickness, Z-potential, hydrodynamic diameter. EU: eudragit; NA: information not available. ^b^ The lowest dose (µg/mL) at which positive results are reported is indicated in brackets. ^c^ The evaluation of cytotoxicity has resulted in a reinterpretation of the authors’ results, based on the recommended 80% cell viability for non-toxic concentrations. Every cytotoxicity assay has been taken into account, regardless of whether it was or was not a proliferation assay; the lowest dose (µg/mL) at which positive results are reported is indicated in brackets. ^d^ Material evaluated by the Fpg-modified version of the comet assay. ^e^ Cellular uptake was assessed. ^f^ Cellular uptake was confirmed. PBMCs: peripheral blood mononuclear cells.

Four of the articles studied genotoxicity by DNA fragmentation. The method consists of isolating cells previously treated with GBMs, extracting genomic DNA, and running samples in gel electrophoresis. Lu et al. [59] used DNA fragmentation to study genotoxicity in mouse embryonic NIH3T3 fibroblasts and human colon cancer HCT116 cells after treatment with nine different GBMs (100 μg/mL, 72 h). No signs of DNA fragmentation were observed. Conversely, Hashemi and colleagues [88] found broken and smeared DNA on mouse embryonic fibroblast (MEF) cells treated with nano- and microsized GO (200 μg/mL). The assay performed in L929 fibroblast and MCF-7 breast cancer cell lines exposed to GO-Fe_3_O_4_ (3.125–100 μg/mL, 48 h) did not exhibit DNA fragmentation [66]. On the other hand, Martinez et al. [69] used flow cytometry to investigate genotoxicity in HeLa, L929, and monocytes exposed to GO (1–24 μg/mL, 24 h). Data extrapolated from side scatter (SSC), namely granularity of the cells, revealed significant DNA fragmentation in HeLa and monocytic cells, in comparison to the negative control.

#### 3.2.4. Other in Vitro Assays

Several studies have assessed GBM-induced DNA damage in bacteria. The mutagenic capacity of different types of GO [89], graphene quantum dots (GQDs) [90], and exfoliated graphene [91], as well as graphene-coated cobalt–chromium discs [92], have been assessed using the validated bacterial gene mutation assay or Ames test (OECD TG 471) [93]. All the tested materials reported negative results, except GQDs displaying a clear dose-dependent response in the *Salmonella typhimurium* strains TA102 and TA104. This pair of strains can detect A:T to G:C substitutions in the DNA molecule produced because of oxidative DNA damage. Interestingly enough, the same GQDs were not mutagenic in the TA98 and TA100 strains, which can detect frameshift mutations and G:C to A:T substitutions, respectively [90]. TA98 and TA100 were also used with all the other materials reporting negative results. In addition, negative results were also obtained with the bacterial strains TA1535 and TA1537, and with the *Escherichia coli* mutant WP2uvr, in the case of exfoliated graphene [91].

The Ames test was already proposed more than a decade ago to be included into a core in vitro battery of assays for assessing mutagenicity of chemicals [94]. However, in the case of nanomaterials, the Ames test may not be a suitable method as some nanomaterials may not be able to penetrate through the bacterial wall, whereas others may kill the bacteria due to their bactericidal effects [23,26,28,43]. In fact, GO nanosheets have been reported to damage the *E. coli* genome after growing the bacteria in the presence of different concentrations of GO solutions (0.004–1 µg/µL). The authors suggested that disbanding of the bacterial genome was produced by the material after entering into the bacteria by puncturing the cell walls [95]. 

Naked DNA was exposed to GBMs to determine the induction of DNA cleavage or mutations. Plasmid pBR^322^ DNA was incubated with N-doped GQDs (0.312–40 μM, 3 h) [84] or Gr@Fe_3_O_4_ NCs (1.33 mg/mL, 2 h) [86] to perform the plasmid nicking assay. After the electrophoretic separation of the DNA, oxidative and hydrolytic reactions in the DNA were found to be induced by N-doped GQDs [84], while no damaging effects were found in Khan et al.’s study [86]. The mutagenic effect of GO was assayed by mixing plasmid DNA containing the gene protein kinase Cζ (PKCζ) and the material in a PCR reaction. After cloning and sequencing the PCR products, results showed a significant increase in the mutation rate induced by GO [60]. These methods can give some insights about GBMs–DNA direct interplays, but they rely on the exposure of isolated DNA. Thus, the lack of cellular membrane, as well as the absence of the repair systems present in the cells, make these methods unsuitable for genotoxicity assessment.

### 3.3. In Vivo Studies

Thirteen studies, published in seven papers, assessed the in vivo genotoxicity of graphene (Table 7). Among them, ten studied the effect of GO [60,96,97,98,99], two of rGO [97,100] and one of exfoliated graphene [91]. Regarding graphene material characterization, it was not found in two of the publications; in the others, the amount of information was not the same among different studies but, in general, lateral size, z-potential, and hydrodynamic diameter are provided. 

Among the ten studies that evaluated GO, six of them used the comet assay [96,97,98,99], three the MN assay [60,98] and one the CA test [96]. Among the two studies that evaluated the genotoxicity of rGO, one used the MN assay [100] and the other one the comet assay [97]. One study, which evaluates the effect of exfoliated graphene, used the MN assay [91]. The experimental system was predominantly mice of different strains [60,91,96,97,98,99] with only one study carried out in Wistar rats [100]. Male was the predominant selected sex [96,98,99,100], with one study carried out in both sexes [60] and two studies in females [91,97]. The size of each experimental group ranged from five to seven animals/group. The route of administration was oral in six of the studies [98,99], followed by the intravenous route in three studies [60,91,100]. Intratracheal instillation [97] and the intraperitoneal route [96] were used in two studies each.

In all the studies, CA and MN were evaluated in bone marrow [60,91,96,98], except one that used peripheral blood lymphocytes [100]. With respect to the comet assay, DNA damage was evaluated in different tissues: liver (four studies) [97,99], lung (three studies) [96,97], bone marrow (two studies) [98], bronchioalveolar lavage (BAL) liquid (two studies) [97], or brain (two studies) [99].

In the CA study [96], five GO doses (10–500 µg/kg b.w.) were given intraperitoneally once a week for one week, or one or two months. A significant increase in the total number of structural chromosome aberrations in all treated groups versus the vehicle group was observed in a dose- and time-dependent manner; no significant numerical chromosome aberrations were observed. In the same study, lung samples of all the animals were taken to carry out the standard comet assay. A significant increase in the % DNA in tail was observed in all treated groups with respect to the vehicle [96]. In this study, lung histopathology revealed some tissue alterations that were more severe after 28 or 56 days of treatment. Moreover, some oxidative stress indicators, such as superoxide dismutase (SOD) and catalase (CAT) activities, reduced glutathione (GSH) content and malondialdehyde (MDA) levels were affected in lung tissue at several doses and time treatments.

In the MN study by Liu et al. [60], a significant dose-dependent increase in the MN frequency of bone marrow polychromatic erythrocytes (PCE) was observed after administrating 1–4 mg/kg b.w. of GO by the intravenous route for five days. GO was not cytotoxic to erythrocytes at any tested dose. Mohamed et al. [98] also observed a significant dose-dependent induction of micronucleated PCE after administrating GO (10–40 mg/kg b.w.) for one or five consecutive days by the oral route. Moreover, they found a dose-dependent increase in DNA damage, expressed as % DNA in tail, in the bone marrow. These authors also obtained samples of the liver and brain from the same animals observing a significant dose-dependent DNA damage increase in both tissues [99]. MDA and GSH level, as well as glutathione peroxidase (Gpx) activity, were measured in both tissues. A dose-dependent MDA increase, and GSH and Gpx decrease, were observed in liver and brain tissues after acute and subacute treatment [99]. On the other hand, histological alterations were observed in the liver and brain of treated animals compared to control animals, ranging from slight to moderate alterations at increasing doses.

Bengston et al. [97] administered GO or rGO at a single dose of 18, 54, or 162 µg by the intratracheal instillation route. Samples of liver, lung and BAL fluid were obtained after 1-, 3-, 28-, or 90-days post-administration, and the standard comet assay was performed. Negative results were obtained in the liver and lungs. In BAL, significantly increased levels of DNA strand breaks were induced by 18 µg of GO at days 3 and 28, 18 µg of rGO at days 1 and 90, and 54 µg of rGO at day 1, when compared to the vehicle groups. A severe acute inflammatory response was observed after a few days of treatments with GO and rGO.

Mendonça et al. [100] did not find an increase in MN frequency in the peripheral blood lymphocytes of Wistar male rats 7 days after a single i.v. administration of rGO (7 mg/kg b.w.). The thiobarbituric acid reactive substances were measured as an indicator of lipid peroxidation. In addition, the oxidation status was evaluated by CAT and SOD activities. No effects on lipid peroxidation or CAT activities were detected, but SOD activity rose progressively from 15 min to 7 days, indicating increased oxidative stress.

The study of Fujita et al. [91] is the only one that carried out a MN test following the OECD TG 474. They administered three doses of exfoliated graphene (0.5, 1, 2 mg/kg b.w.) to mice by the i.v. route during five consecutive days. They found no statistically significant increase in MN frequency and no significant decrease in the ratio between PCE and normochromic erythrocytes. However, the presence of exfoliated graphene in the bone marrow was not confirmed. 

Some articles were retrieved in the bibliographic search, but they have not been included in Table 7 because their main objective was not the study of the genotoxicity of GBMs. Priyadarsini et al. [101] demonstrated teratogenic effects of GO nanosheets in *Drosophila melanogaster*; however, apart from effects on the development, they carried out the comet assay in the hemocyte cells of the flies’ hemolymph and detected increased DNA damage. Oliveira et al. [102] studied the biocompatibility and toxicity of a nanomaterial composed of graphene nanoribbons and nanohydroxyapatite, which are used as regenerative scaffolds in an in vivo osteopenia model. The standard comet assay was carried out in blood cells, bone marrow, and the liver of animals implanted with the graphene biomaterial and negative results were obtained in all conditions. Zambrano-Andazol et al. [56] studied the biocompatibility and genotoxicity of rGO membranes (rGOM) intended to be used in ocular regenerative medicine. The in vivo rGOM genotoxicity was studied by the standard comet assay performed in the liver tissue of Wistar rats used for the in vivo biocompatibility assay. No statistically significant differences in the percentages of DNA in tail were found between rats transplanted with or without rGOM.

### 3.4. Other Studies

Two of the selected articles used in silico approaches to evaluate the potential genotoxicity of GBMs [103,104]. The model developed by Kong and colleagues [103] allowed them to find a relationship between the size of the GQDs and the DNA damaging mechanisms. The small GQDs intercalated into the DNA molecule and caused DNA base mismatch, whereas the large GQDs linked to the two ends of the DNA molecule and caused DNA unwinding. On the other hand, in silico analysis using the functional density theory demonstrated that unoxidized graphene is unable to generate ROS, but it could link with the DNA bases by covalent and non-covalent bonds. Conversely, GO induced ROS-mediated genotoxicity [104]. Interestingly, both studies agreed on a higher affinity of GBMs to interact with guanine than with the other DNA bases. 

Only one human biomonitoring study was retrieved by our literature search [105]. Workers unintentionally exposed to FLG (average lateral size ~1.15 µm, thickness 1.6 nm) during the production process by liquid-phase exfoliation were monitored for different biomarkers of effects. The induction of micronuclei in buccal cells and DNA damage in lymphocytes (comet assay), presence of oxidized DNA bases (8-oxoGua, 8-oxoGuo and 8-oxodGuo) in urine, as well as biomarkers of oxidative stress in exhaled breath condensate and inflammation (cytokines release) in serum were assessed in six workers. Another six workers producing silica nanoparticles were included in the same study. Eleven unexposed workers served as the negative control group. There was an increase of MN frequency in respect to controls for both the groups of workers (graphene and silica), although the differences were not statistically significant due to the small group sizes. Despite exposure to FLG, DNA damage levels in lymphocytes did not increase as measured by the standard comet assay, it showed a significant increase of oxidative DNA damage (measured by the Fpg-modified comet assay). No differences between FLG workers and controls could be found for the oxidative stress or inflammatory biomarkers. 

## 4. Identification of Gaps and Recommendations

The GBMs retrieved in the current review were classified into different sub-families according to their PC characteristics, as previously recommended [2,6,7]. GO of different type and functionalization was the most studied GBM for genotoxicity, as previously reported for other toxicity endpoints [5]. rGO was the second most tested GBM depending on the assays performed. However, there was a huge diversity of materials, even within each category. Furthermore, GBMs are often poorly PC characterized [2,5], which together with the broad variability of biological systems and exposure conditions used precludes the identification of PC properties that could drive the genotoxicity response. In the present review, the dimensions of the studied material were usually provided. Nevertheless, other relevant toxicity-related properties, such as the oxidation state or the presence of impurities, were not systematically analyzed. Therefore, it is highly recommended that a complete PC characterization of the GBMs is carried out when assessing the genotoxic potential of these materials in the future.

Most of the genotoxicity studies with GBMs were carried out using in vitro approaches, which can only identify primary mechanisms of action. However, primary direct and indirect mechanisms of action are not easily distinguished in these approaches. According to the outcomes of the in silico models and the results from the DNA fragmentation assays, GBMs would be able to directly react with and damage the DNA molecule if the material came in direct contact with it. GQDs have been reported to enter the nucleus and directly interact with DNA [106]. The authors observed cleavage and cross-linking of DNA strands, which could be induced by direct contact via H-bonding and π-π stacking, but also through indirect mechanisms (e.g., ROS formation). The hypothetical penetration of rGOs into the nucleus and their direct contact with DNA was concluded by Akhavan and colleagues [73] when no ROS production or RNA efflux increase were observed, while positive genotoxic responses were induced after testing low concentrations. This effect was experimentally verified a year later by evidencing rGONPs’ penetration into the nucleus through confocal fluorescence imaging [74]. As DNA-reactive substances are assumed not to have a threshold response [107], further clarification of the capacity of GBMs to directly react with the DNA molecule is extremely important in establishing occupational exposure limits for GBMs.

As in the case of other nanomaterials, oxidative stress mediated by ROS generation is one of the main indirect mechanisms of GBMs’ genotoxicity [29]. For instance, ROS-dependent DNA damage (detected by the comet assay) was observed in human retinal pigment epithelium ARPE-19 cells after 24 h exposure to GO and rGO [68]. The results obtained with the enzyme-modified version of the comet assay can provide some insights into the role of oxidative DNA damage [23,38]. However, only one in vitro study [70] and the single biomonitoring study [105] identified in the present review performed such assay. Thus, we recommend the use of enzymes to detect oxidized bases when applying the comet assay in genotoxicity testing of GBMs.

As a consequence of the few available studies using whole organisms, the involvement of secondary mechanisms on GBMs’ genotoxicity has been poorly studied. Studies in rodents after exposure to GBMs by the respiratory route (inhalation, intratracheal instillation or pharyngeal aspiration) revealed relatively severe lung inflammation [2], which could trigger a genotoxic response. One of the studies retrieved in the present review used an in vitro co-culture system composed of human-transformed type-I alveolar epithelial cells (TT1) and differentiated THP-1 monocytes (macrophages) [64]. While results showed significant MN induction after the exposure of the model to FLG materials (neutral, aminated, and carboxylated), a pre-treatment with the antioxidant N-acetylcysteine reduced the genotoxicity to baseline levels. These findings revealed the potential of these FLG materials to promote secondary mechanisms of DNA damage, probably oxidative stress, under the studied conditions. As a threshold mode-of-action is assumed when genotoxicity is mediated by secondary mechanisms [107], such distinction is highly relevant for the risk assessment of GBMs. 

From a regulatory perspective, the mutagenicity assessment of GBMs should be performed by using a battery of in vitro validated assays, which can be followed up by the corresponding validated in vivo tests, depending on the in vitro outcome [23,50]. The capacity to induce both gene mutations, as well as structural and numerical chromosomal alterations, should be evaluated. As reflected in Table 6, only two publications reported results with the mammalian gene mutation assay for GNPs [80] and oxygen functionalized graphene [58], making it impossible to raise conclusions on the capacity of GBMs to induce this type of damage. Having in mind the lack of suitability of the bacterial mutation assays for nanomaterials, mammalian gene mutations assays should be included in the evaluation of any nanomaterial [26] to allow them to fit to safety requirements before being launched to the market. 

Based on the outcomes of the evaluations carried out with the MN and CA, GBMs seem to be able to induce chromosomal damage. CA was assessed in four in vitro studies [58,73,74,79] and one in vivo study [96]. All of them focused on determining structural aberrations; however, no data regarding the potential of GBMs to induce numerical aberrations was available. On the other hand, only one study combined the analyses of MN with centromeric staining by FISH techniques, which allows for the detection of chromosome losses [63]. Studies such as the latter one are highly recommended to distinguish between the aneugenic and clastogenic capacities of GBMs. 

Despite inhalation being the main exposure route to GBMs in occupational settings [2], only one in vivo study was performed by intratracheal instillation of GO and rGO [97]. No systemic (liver) genotoxicity was observed in this study, nor was it in the other in vivo studies reporting lack of induction of MN by GBMs, whose target tissue is the bone marrow [91,100]. One main concern with nanomaterials is whether they can reach the target tissues when using treatment schedules that have been optimized for soluble chemicals [16]. Although guidelines to assess the toxicokinetics of nanomaterials are still in progress [108], future in vivo genotoxicity studies with GBMs should confirm the presence of the material in the target tissue, especially if the outcomes are negative.

Human population monitoring studies can offer highly relevant toxicological information. However, few studies are available for nanomaterials, as these types of studies are challenging to conduct [16]. Only one study of insufficient sample size was retrieved in the present review [105]. In addition, limited information on airborne GBM concentrations in occupational settings is currently available [2]. Properly designed biomonitoring studies of GBMs should be conducted, and the results should be correlated with those provided by the in vitro approaches to gain insight into the mechanisms of action operating in potential genotoxic responses observed in humans exposed to GBMs.

## Figures and Tables

**Table 1:** In vitro studies assessing the genotoxicity of GBMs using the micronucleus assay.

Material	Characterization ^a^	Cell Line	Dose Range (µg/mL) and Treatment Time	Result ^b^	Cytotoxicity ^c^	Reference
GO	NA	Human fibroblasts	10	Negative	Negative	[78]
GO nanosheets	20 µm	JURKAT	6.25–400 (24 h)	Negative ^d^	Positive (400)	[65]
	NA	WIL2-NS		Negative ^d^	Negative	
	−21 mV					
	8276 nm	Human primary lymphocytes		Positive (50)	Positive (400)	
GO-Fe_3_O_4_	NA	L929	3.125–100 (48 h)	Negative	Negative	[66]
		MCF-7			Negative	
Oxygen functionalized graphene	<10 μm	TK6	10–100 (4 h)	Negative ^d,e^	Negative	[58]
	1–1.2 nm					
	−64.66 ± 9.83 mV	NH32				
	237.1 ± 17.2 nm					
Neutral few-layer graphene	153.2 ± 19.2 nm	16HBE14o−	2–100 (24 h)	Positive (10)	Negative	[63]
	94.73 ± 67.94 nm	TT1	2–100 (24 h)	Positive (20)	Negative	[64]
	−31.72 ± 1.95 mV					
	290.8 ± 302.6 nm	TT1/d.THP-1	4–50 (24 h)	Positive (10)		
Aminated few-layer graphene	163.8 ± 21.7 nm	16HBE14o−	2–100 (24 h)	Positive (50)	Negative	[63]
	86.20 ± 42.16 nm	TT1	2–100 (24 h)	Positive (8)	Negative	[64]
	−41.96 ± 0.86 mV					
	170.1 ± 97.92 nm	TT1/d.THP-1	4–50 (24 h)	Positive (10)		
Carboxylated few-layer graphene	158.5 ± 19.6 nm	16HBE14o−	2–100 (24 h)	Negative ^d,e^	Negative	[63]
	55.16 ± 42.22 nm	TT1	2–100 (24 h)	Positive (8)	Negative	[64]
	−34.36 ± 3.06 mV					
	169.6 ± 76.88 nm	TT1/d.THP-1	4–50 (24 h)	Positive (20)		

^a^ Characterization of GBM is indicated as follows: lateral size, thickness, Z-potential, hydrodynamic diameter. NA: information not available. ^b^ The lowest dose (µg/mL) at which positive results are reported is indicated in brackets. ^c^ The evaluation of cytotoxicity has resulted in a reinterpretation of the authors’ results, based on the recommended 55 ± 5% cytotoxicity limit established in the OECD TG 487 [42] for non-toxic concentrations. Every cytotoxicity assay has been considered, regardless of whether it was not a proliferation assay recommended by the OECD TG 487 [42]; the lowest dose (µg/mL) at which positive results are reported is indicated in brackets. ^d^ Cellular uptake was assessed. ^e^ Cellular uptake was confirmed.

**Table 7:** In vivo studies assessing GBMs genotoxicity.

Material	Characterization ^a^	Assay (Tissue Evaluated)	Experimental System (Strain; Sex)	Doses (Route of Administration)	Treatment Schedule (Sampling Time)	Results	Ref.
GO	NA	CA (BM)	Mice (Albino; males)	10, 50, 100, 250, 500 μg/kg b.w. (i.p.)	1 per week for 7, 28, 56 days (NA)	Positive	[96]
		Comet (Lung)				Positive	
GO	NA	MN (BM)	Mice (Kunming; males and females)	1, 2, 4 mg/kg b.w. (i.v.)	5 days (NA)	Positive	[60]
GO	62.5 ± 51.42 nm NA −37.1 mV 1162 nm	MN (BM)	Mice (Swiss; males)	10, 20, 40 mg/kg b.w. (oral)	1 dose (24 h)	Positive	[98]
					Daily, 5 days (24 h)	Positive	
GO	62.5 ± 51.42 nm NA	Comet (BM)	Mice (Swiss; males)	10, 20, 40 mg/kg b.w. (oral)	1 dose (24 h)	Positive	[98]
	−37.1 mV						
	1162 nm				Daily, 5 days (24 h)	Positive	
GO	2–3 µm NA −49.7 mV 199–625 nm	Comet	Mice (C57BL/6J; females)	18, 54, 162 μg/mouse (i.t.)	1 dose (1, 3,28, 90 days)		[97]
		(BAL)				Positive	
		(Lung)				Negative ^b^	
		(Liver)				Negative ^c^	
GO	62.5 ± 51.42 nm NA −37.1 mV 1162 nm	Comet (Liver)	Mice (Swiss; males)	10, 20, 40 mg/kg b.w.(oral)	1 dose (24 h)	Positive	[99]
					Daily, 5 days (24 h)	Positive	
		Comet (Brain)			1 dose (24 h)	Positive	
					Daily, 5 days (24 h)	Positive	
rGO	342 ± 23.5 nm 5 nm	MN (PBL)	Rats (Wistar; males)	7 mg/kg b.w. (i.v.)	1 dose (7 days)	Negative	[100]
	−23 ± 0.18 mV NA						
rGO	1–2 μm NA −13.9 mV 250–271 nm	Comet (BAL) (Lung) (Liver)	Mice (C57BL/6J; females)	18, 54, 162 μg/mouse (i.t.)	1 dose (1, 3, 28, 90 days)		[97]
						Positive	
						Negative ^b^	
						Negative ^c^	
Exfoliated graphene	NA NA NA 1–40 µm	MN (BM)	Mice (ICR; males)	0.5, 1, 2 mg/kg b.w. (i.v.)	Daily, 5 days (NA)	Negative ^c^	[91]

^a^ Characterization of GBM is indicated as follows: lateral size, thickness, Z-potential, hydrodynamic diameter; NA: information not available; CA: chromosome aberrations; MN: micronucleus; BM: bone marrow; PBL: peripheral blood lymphocytes; BAL: bronchoalveolar lavage; i.p.: intraperitoneal; i.v.: intravenous; i.t.: intratracheal instillation. ^b^ The presence of the test compound was confirmed in these tissues. ^c^ The presence of the test compound was not studied in these organs.
